# Pandemic airway management: A cognitive aid to increase safety and team cohesion during intubation, donning, and doffing

**DOI:** 10.1177/1751143720931614

**Published:** 2020-05-25

**Authors:** Peter G Brindley, Jarrod M Mosier, Christopher M Hicks

**Affiliations:** 1 Department of Critical Care Medicine, Department of Anesthesiology and Pain Medicine and the Dosseter Ethics Centre, University of Alberta, Edmonton, Canada; 2 Department of Emergency Medicine, Division of Pulmonary, Allergy, Critical Care and Sleep, University of Arizona College of Medicine, Tucson, AZ, USA; 3 Department of Medicine, Division of Pulmonary, Allergy, Critical Care and Sleep, University of Arizona College of Medicine, Tucson, AZ, USA; 4 Department of Emergency Medicine, St Michael's Hospital, University of Toronto, Ontario, Canada

Viruses such as the severe acute respiratory syndrome coronavirus 2 (SARS-CoV-2), which causes the Coronavirus disease 2019 (COVID-19), can create performance-retarding anxiety and information-overload; especially for stressful procedures like intubation, and when there are unfamiliar steps. We offer this simple mnemonic/checklist/cognitive aid utilizing the five letters: C.O.V.I.D. Our goal is to allay fears, expedite action, decrease viral spread, and highlight what has changed. This can be used for any highly infectious aerosol-generating viruses, or whenever enhanced personal protective equipment (PPE) is required.

An easy-to-remember cognitive aid may help enhance shared mental models (especially if PPE impairs communication), maintain cognitive bandwidth (via a common aide-memoire), increase safety (by decreasing time in infected rooms, increasing first-pass success, and optimizing donning and doffing) and help cross-monitor team members via “buddy checks”.

Even without COVID-19, airway management is more dangerous and complex when performed away from operating rooms, and using unfamiliar staff.^1^ Cognitive aids with fewer than seven steps and which ask questions (i.e. “what will you do, and when”) appear superior to those that are longer or passive.^2^ Moreover, checklists should facilitate teamwork and not just individual taskwork.^3^

Pandemic airway management can mean undoing years of muscle memory (e.g. avoiding bagging, high flows, to prevent aerosolization). Moreover, much prior work regarding airway management focused on anatomical difficult airways, or physiologically difficult airways (i.e. low blood pressure, right ventricular pathology).^4^ While both are important, our new reality means increased attention to situational difficulty (personal fear, situational unfamiliarity).^1–5^ Because of the increased need for coordination, role clarity, and shared safety, we offer a simple five-step acronym using five letters that none of us will forget.

Step 1: C-
  **Coordinate** who will do what and when. Perform a pre-brief^3^ where roles are assigned before entering the room, and assign “buddies” to check that PPEs offer body coverage.
  **Collect** all equipment at bedside, so that you do not have to doff and leave room.
  **Colleague** outside of the room. Available to help if needed and already wearing PPE.
Step 2: O-
  **Only** have three people in the room and use the most experienced intubator and techniques that increase first pass success (i.e. full-dose paralysis).
  **Outside the room** until your PPE has been checked by your buddy, and negative pressure turned on (if available).
  **Obstruct** the endotracheal tube (ETT) with a clamp prior to connecting the ventilator.
Step 3: V-
  **Videolaryngoscopy** is preferable to decrease the intubator's exposure to aerosols.
  **Voice communication** with those outside the room (activate a microphone or walkie-talkie)
  **Verify** tube placement with ETCO_2_ and that the ETT cuff is inflated before initiating positive pressure breaths.
Step 4: I-
  **Inflate** the ETT cuff prior to bagging or placement on the ventilator.
  **Interrupt** the circuit as infrequently as possible and only at end expiration.
  **Insert** a supraglottic airway rather than using vigorous bag-mask ventilation.
Step 5: D-
  **Don and Doff** safely (include a buddy check and 15-s hand-washing whenever gloves, gowns or masks are touched).
  **Double glove** (intubator only) and apply sanitizer to outside of soiled gloves before removal.
  **Don't leave the room prematurely**, i.e. before your buddy has given the “okay”.

## In closing

While this mnemonic has not been tested empirically, it received iterative multi-professional input (MD, RN) and multidisciplinary input (Critical Care, Emergency Medicine, Anesthesia). It was finessed during 10 drafts and 20 high-fidelity mannikin simulations, until no further changes were requested. It was deemed robust enough to work throughout the hospital, and was associated with increased subjective team safety, team cohesion, and esprit de corps.
